# Safety Profile of Probiotics as an Adjuvant to Oral Immunotherapy for Food Allergies: A Meta Analysis of Randomized Controlled Trials

**DOI:** 10.1007/s12602-025-10544-z

**Published:** 2025-05-05

**Authors:** Mirna Hussein, Safia Essam, Mennatullah Essam Mekky, Lujaina Ahmed, Heidi Sherif Farouk, Batool Sami Alshanshoury, Jana Gado, Hassan El-Masry, Hamed Gaber

**Affiliations:** Faculty of Medicine, Alexandria University, Khartoum Square, Alexandria, 21526 Egypt

**Keywords:** Food allergy, Probiotics, Oral immunotherapy

## Abstract

Food allergies are common, affecting a sizable portion of children and adults in the Western world. In recent years, a promising treatment known as allergen immunotherapy (AIT) has emerged. This technique aims to address the fundamental immune system dysfunction that underlies food allergies. The study's objective was to evaluate the safety of combining probiotics with AIT, compared to placebo in treating patients with food allergies. A comprehensive literature search in several databases was conducted. All included studies were randomized controlled trials(RCTs). Meta-analyses were performed using R software. Statistical tests to assess safety outcomes and risk of bias were used. After reviewing 519 studies, three studies were included in the systematic review and meta-analysis, involving a total of 258 patients (141 in the probiotics group, 144 in the placebo group). The evaluation of the safety profile of the integrated probiotic therapy revealed an overall favorable outcome, with no significant differences in adverse events compared to placebo for most organ systems examined. However, a potential increase in oral adverse effects was observed in the probiotics group (RR = 1.7573, 95% CI: 0.3941 to 7.8359), although this result did not reach statistical significance. The combination of probiotics and allergen-specific immunotherapy appears to be generally safe and shows promise as a valuable treatment approach.

**Supplementary Information:**

The online version contains supplementary material available at 10.1007/s12602-025-10544-z.

## Introduction

Globally, food allergies affect 5% of adults and 8% of children in the Western world [1, 2]. Current main approaches to food allergy therapy target side-effect treatment with antihistamines and allergen avoidance [3]. While effective, allergen avoidance may be challenging for patients, particularly for those diagnosed with multiple food allergies. Allergen immunotherapy (AIT) has emerged in recent years as a promising therapeutic strategy that addresses the immunological dysregulation associated with food allergies [4, 5].

AIT may be considered in patients with severe food allergies, or in patients where food allergies exert a significant impact on the quality of life. Current allergen immunotherapies target the following food allergens: cow's milk, eggs, and peanuts [6, 7]. The immunotherapy may be administered orally, sublingually, subcutaneously, and epicutaneously. Numerous studies have suggested that oral immunotherapy (OIT) and epicutaneous immunotherapy are more effective than sublingual and subcutaneous immunotherapy. On the other hand, oral immunotherapy has a greater rate of side effects than epicutaneous [8–10]. Nevertheless, significant concerns remain regarding the safety of AIT, and its ability to induce permanent tolerance.

Probiotics have gained recognition as a solo or adjuvant therapy for numerous medical conditions, due to their immunomodulatory effects and potential to adjust the intestine microbiota composition. In food allergy therapy, probiotics have been included as an adjuvant to AIT, to increase desensitization and decrease adverse effects. Lactobacilli has been used in most studies investigating probiotics as an adjuvant to immunotherapy [11]. Ren et al. observed that *Bifidobacterium* *breve* alleviated allergic rhinitis symptoms by modulating immune responses, particularly by enhancing regulatory T cell activity [12]. In addition, a study conducted by Chen et al. found that* Lactobacillus Plantarum* improved allergic rhinitis symptoms in mice by reducing airway inflammation and altering cytokine levels [13]. Another study found that *clostridium butyricum* administration reduced airway inflammation and rebalanced Th1/Th2 immune responses in asthmatic mice [14]. Kim et al. demonstrated that a probiotic mixture alleviated allergic rhinitis by rebalancing Th2/Treg cells and restoring gut microbiota balance [15]. However, limited data is available on the efficacy and safety of probiotics as an adjuvant to AIT for the treatment of food allergies.

In this systematic review and meta-analysis, we aim to evaluate the safety of combined probiotics and AIT compared to placebo. Furthermore, we will critically assess the existing body of evidence from preclinical and clinical trials to give light on the safety and potential limitations of this integrated therapy strategy.

## Methods

### Search Strategy and Screening

This study adhered to the Preferred Reporting Items for Systematic Reviews and Meta-Analyses (PRISMA) guidelines. Two independent investigators (SE and MH) conducted a thorough search of the literature in the Cochrane Library, PubMed, Scopus, and Web of Science databases, using the following terms: “probiotics,” “allergy immunotherapy,” “food allergies,” “treatment.” (Supplemental File 1) The reference lists of retrieved articles were also searched. The studies included were those with patients diagnosed with food allergies, receiving allergen-specific immunotherapy with probiotics, published from database inception to January 2024. The two authors performed a title and abstract screening and full-text review of the resulting entries using Covidence ®; inclusion decision conflicts in both stages were solved by a third party (LA) [16]. After screening and before commencing data extraction, the review was registered in PROSPERO (ID number: CRD42024545635).

### Article Selection

The inclusion criteria for articles in this systematic review and meta-analysis were as follows: studies published from database inception to 2024, studies conducted in pediatric patients diagnosed with food allergies, studies with allergen-specific immunotherapy (AIT) and probiotics as the treatment of interest, and studies with at least one comparison group. On the other hand, studies conducted in patients diagnosed with other atopic diseases, and using other interventions, and using either AIT or probiotics as the treatment of interest were excluded, as were papers written in other languages and animal studies. Studies that did not use a randomized, double-blinded, placebo-controlled study design were excluded.

### Quality Assessment

To promote transparency and ensure accuracy, two independent investigators conducted the risk of bias assessment. As all the included studies were randomized controlled trials (RCTs), the Cochrane Risk of Bias (RoB) Tool was used to evaluate them [17]. The Cochrane RoB tool assesses RoB across six domains: allocation concealment, blinding, incomplete outcome data, outcome assessment, random sequence generation, and selective reporting. The RoB results can be found in Supplementary Fig. 1.

### Data Extraction and Measured Outcomes

Two investigators extracted data from the included studies. Key baseline characteristics that were extracted included age, weight, sex, diagnosis of asthma, diagnosis of eczema, anaphylaxis to peanut, and diagnosis of multiple food allergies. The extracted study characteristics were author, date of publication, type of study, length of follow-up, type of intervention, and intervention group. The endpoints of interest in this meta-analysis were the incidences of gastrointestinal (GIT) adverse effects, skin adverse effects, urticaria, upper respiratory adverse effects, lower respiratory adverse effects, oral adverse effects, and serious adverse events (anaphylaxis).

### Data Analysis

Meta-analyses were conducted using the R software version 4.3.3, by using the meta package. For the evaluation of dichotomous outcomes, the risk ratio (RR) was employed, accompanied by 95% confidence intervals (Cis) to quantify the precision of the estimates. To gauge the degree of heterogeneity among the amalgamated studies, we employed both the Chi-square test and the I-square test. A consensus of heterogeneity was established when the Chi-square test yielded a p-value of less than 0.05, concurrently with an I-square value surpassing 50%. In instances where the pooled studies exhibited homogeneity, a fixed-effect model was applied to synthesize the data cohesively. Conversely, for situations characterized by significant heterogeneity, a random-effects model was invoked to aggregate the data, acknowledging the potential variability across studies. Egger test was used to assess the funnel plots asymmetry.

## Results

### Search and Selection

**Fig. 1:**
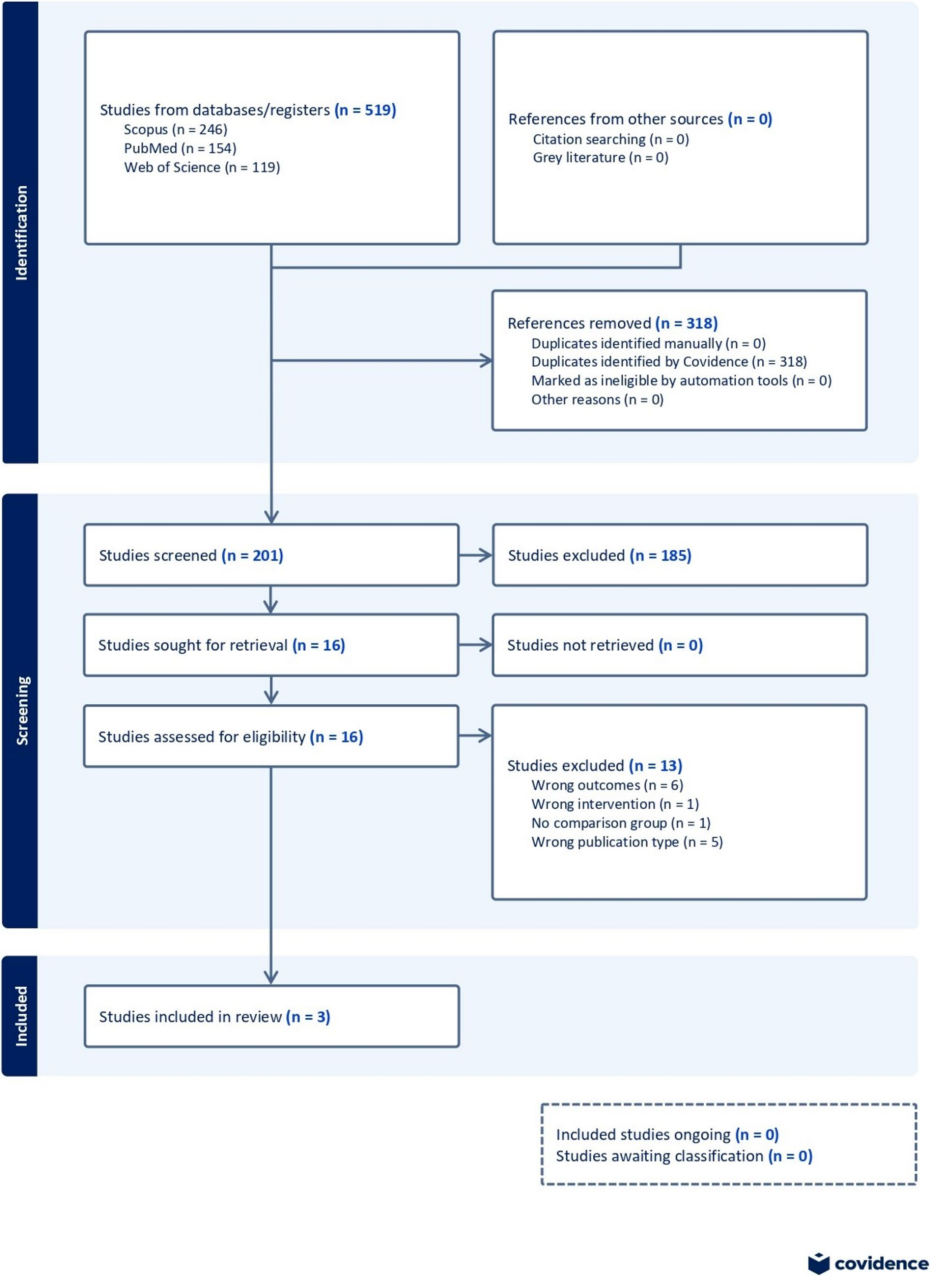
A PRISMA Flowchart detailing the search and selection process

The PRISMA Flowchart in Fig. 1 summarized the search and selection process of this study. The initial literature search yielded 519 results, with 318 total duplicates removed by Covidence. At the end of the title and abstract screening, 188 studies were excluded, leaving 13 studies in the full-text review. The remaining studies were thoroughly assessed, with 10 studies ultimately excluded. A total of three studies were included in the systematic review and meta-analysis [18–20]. (Fig. 1).

### Study Characteristics

The characteristics of the included studies are outlined in Supplemental Table 1. The studies included in the meta-analysis were published between 2015 and 2022. 258 patients were included, with 141 in the probiotics group and 144 in the placebo group.

In two of the three included studies (Yamamoto et al. and Loke et al.), the comparator group included in the meta-analysis received oral immunotherapy with placebo probiotics [19, 20]. In the third study included (Tang et al.), the comparator group received placebo immunotherapy with placebo probiotics [18]. (Supplemental Table 1).

## Patient Characteristics

Supplemental Tables 2 and 3 summarize the patients’ baseline characteristics. The included studies of the analysis were published between January 2015 and February 2023. In total, approximately 285 patients were included, with 141 in the probiotics group, and 144 in the placebo probiotics group. Among all the included participants, 103 (36.1%) were female and 182 (63.9%) were male. The average age of the patients was 5.7 years. Among the included patients, approximately 212 (74.4%) were previously diagnosed with eczema, 105 (36.8%) had asthma, and 154 (69.1%) had multiple food allergies. Among the peanut allergy cohort, 84 patients (37.5%) had a history of anaphylaxis to peanuts, and their median peanut-specific IgE levels were 10.9.

Among the 141 participants in the probiotics group, 51 were female, and 90 were male. The average age of participants in the probiotics group was 5.8 years. Approximately 107 (75.9%) were also diagnosed with eczema, 59 (41.8%) had asthma, and 79 (71.8%) had multiple food allergies. Furthermore, 42 (38.2%) peanut allergy patients in the probiotics group had a history of anaphylaxis to peanuts, and their median peanut-specific IgE levels were 11.50. The 144 participants in the placebo probiotics group also possessed similar demographic distributions, with 52 participants being female, and 92 being male. The average age of participants in the placebo probiotics group was 5.6 years. Approximately 105 (72.9%) had concomitant eczema, 59 (41%) had asthma, and 75 (66.4%) had multiple food allergies. Moreover, the median peanut-specific IgE levels of the peanut allergy patients was 10.3, and 42 (36.8%) patients had a history of anaphylaxis to peanuts. (Supplemental Table 2) (Supplemental Table 3).

## Ongoing Trials

Table 1 summarizes the currently ongoing clinical trials examining the efficacy and safety of probiotics as an adjuvant to allergen-specific immunotherapy. Due to the lack of published data, these trials were not included in the meta-analysis (Table 1).

**Table 1:** Ongoing clinical trials examining the efficacy and safety of probiotics with allergen immunotherapy

Trial Identifier	Trial name	Country	Allergen	Intervention(s)	Expected timeframe	Status
JPRN-jRCTs041220087	Egg oral immunotherapy with probiotics for severe egg allergy [30]	Japan	Egg allergy	Probiotics (0.03 g of LGG + 0.3 g of Bifidobacterium bifidum TMC3115) and heated hen’s egg powder Placebo and heated hen’s egg powder	2023—present	Recruiting
ACTRN12619000480189	Randomised, controlled trial evaluating the effectiveness of probiotic and egg oral immunotherapy at inducing desensitisation or tolerance in participants with egg allergy compared with placebo (Probiotic Egg Allergen Oral Immunotherapy for Treatment of Egg Allergy: PEAT study) [31].	Australia	Egg allergy	Egg white protein mixed into food (excluding food containing eggs) and one standardized scoop of probiotic mixed into water Egg white protein or placebo mixed into food and one standardized scoop of probiotic or placebo mixed into water	2019 – present	Active, not recruiting
NCT05165329	A Randomized, Controlled Trial of Probiotic and Peanut Oral Immunotherapy (PPOIT) in Inducing Tolerance in Hong Kong Children With Peanut Allergy Compared With Oral Immunotherapy (OIT) Alone and With Placebo [32]	Hong Kong	Peanut allergy	Peanut oral immunotherapy (peanut flour with 50%) peanut protein and L. rhamnosus GG probiotic Peanut oral immunotherapy and placebo probiotic Placebo peanut oral immunotherapy (maltodextrin) and placebo probiotic	Nov. 2021 – Dec. 2024	Active, not recruiting
NCT06297083	Analysing HIgh Dose Probiotic Peanut Oral Immunotherapy (PPOIT) and High Dose Peanut Oral Immunotherapy (OIT) Versus LOw Dose Peanut OIT for Peanut Allergy (HILO) [33]	Australia	Peanut allergy	Peanut Oral Powder and Probiotic (LGG®, Lactobacillus Rhamnosus) or placebo probiotic (maltodextrin)	May 2024 – Feb. 2027	Recruiting

## Meta-Analysis Results

### Gastrointestinal Adverse Events

**Fig. 2:**
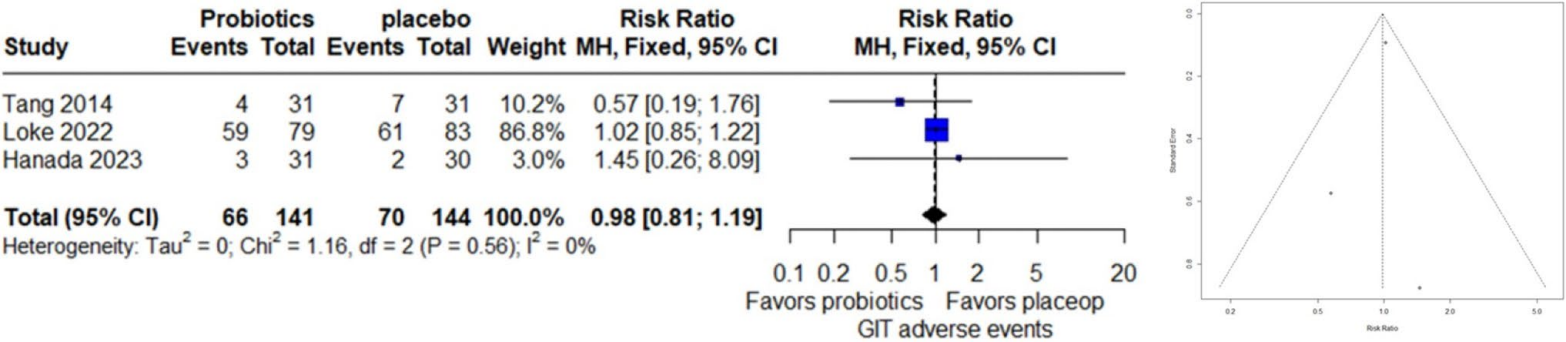
Forest Plot (left) and funnel plot (right) of GIT adverse events

A meta-analysis comparing gastrointestinal (GIT) adverse events between probiotics and placebo included data from 3 studies with 285 observations, noting 136 events. The analysis under the common effect model revealed a relative risk (RR) of 0.9837 (95% CI: 0.8109 to 1.1933), indicating no significant difference in GIT adverse events between probiotics and placebo (z = − 0.17, p = 0.8673). Heterogeneity assessment suggested minimal variability across studies (I^2 = 0.0%, p = 0.5596). Furthermore, the funnel plot asymmetry test found no evidence of publication bias (t = − 0.37, df = 1, p = 0.7739), with a bias estimate of − 0.3113 (SE = 0.8391) (Fig. 2).

### Skin and Subcutaneous Tissue Adverse Events

**Fig. 3:**
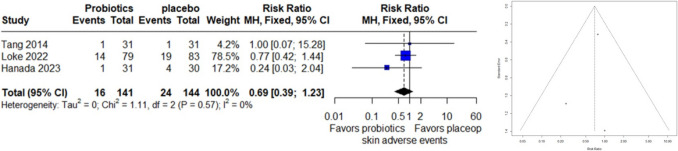
Forest Plot (left) and funnel plot (right) of skin adverse events

A meta-analysis examining skin and subcutaneous tissue adverse events across 3 studies, with a total of 285 observations, reported 40 events. Under the common effect model, the relative risk (RR) was 0.6920 (95% CI: 0.3897 to 1.2290), indicating no significant difference in adverse events between treatments (z = − 1.26, p = 0.2090). Heterogeneity assessment suggested minimal variability across studies (I^2 = 0.0%, p = 0.5733). The funnel plot asymmetry test found no evidence of publication bias (t = − 0.59, df = 1, p = 0.6623), with a bias estimate of − 0.5405 (SE = 0.9215) (Fig. 3).

### Upper Respiratory Tract Adverse Events

**Fig. 4:**
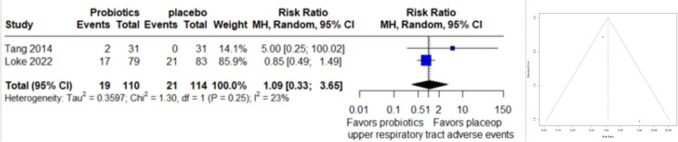
Forest Plot (left) and funnel plot (right) of upper respiratory tract adverse events

The forest plot encompassed data from 2 studies, comprising 224 observations, with 40 reported events related to upper respiratory tract adverse events. Utilizing a random effects model, the calculated relative risk (RR) was 1.0912 (95% CI: 0.3263 to 3.6491), indicating no significant difference in adverse events between treatments (z = 0.14, p = 0.8873). Heterogeneity assessment revealed moderate variability across studies (I^2 = 22.9%, p = 0.2547). Due to the small number of studies, assessment of funnel plot asymmetry by egger test couldn’t be performed (Fig. 4).

### Lower Respiratory Tract Adverse Events

**Fig. 5:**
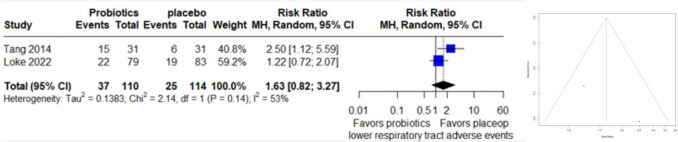
Forest Plot (left) and funnel plot (right) of lower respiratory adverse events

A forest plot comprising data from 2 studies, with 224 observations and 62 reported events related to lower respiratory adverse events, was conducted. Employing a random effects model, the computed relative risk (RR) was 1.6321 (95% CI: 0.8155 to 3.2665), indicating no statistically significant difference in adverse events between treatments (z = 1.38, p = 0.1664). Heterogeneity analysis demonstrated moderate variability across studies (I^2 = 53.3%, p = 0.1433). Due to the small number of studies, assessment of funnel plot asymmetry by egger test couldn’t be performed (Fig. 5).

### Oral Adverse Events

**Fig. 6:**
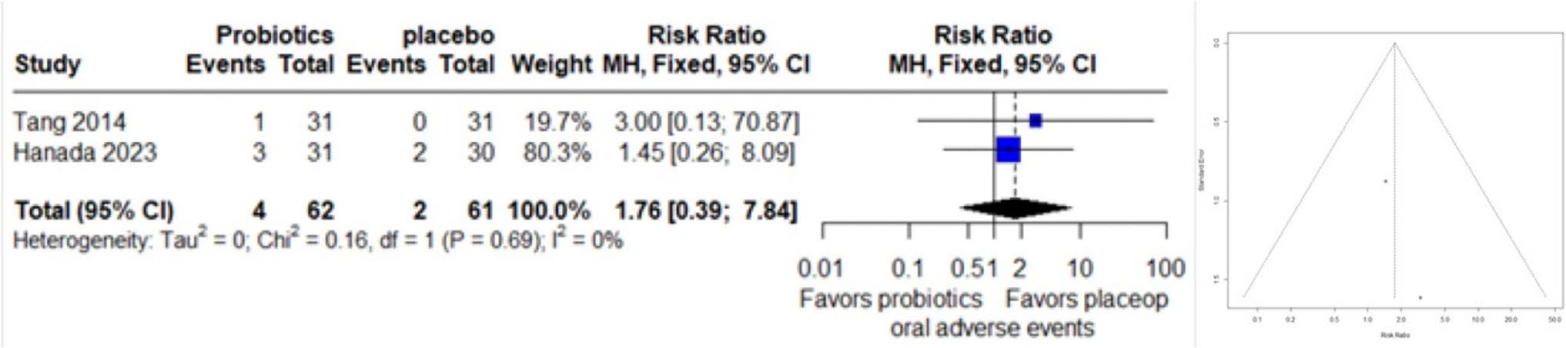
Forest plot (left) and funnel plot (right) of oral adverse events

A forest plot encompassed data from 2 studies, with 123 observations and 6 reported events related to oral adverse effects, was conducted. The common effect model yielded a relative risk (RR) of 1.7573 (95% CI: 0.3941 to 7.8359), indicating a potential increase in adverse effects, although this result did not reach statistical significance (z = 0.74, p = 0.4598). There was no observed heterogeneity between the studies (I^2 = 0.0%, p = 0.6926). Due to the small number of studies, assessment of funnel plot asymmetry by egger test couldn’t be performed (Fig. 6).

### Serious Adverse Events

**Fig. 7:**
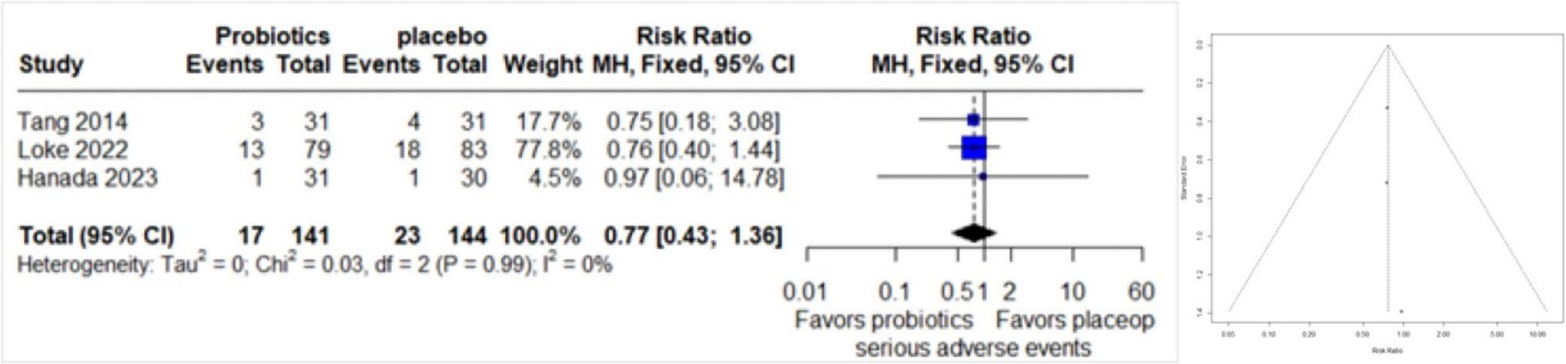
Forest plot (left) and funnel plot (right) of serious adverse events

A forest plot comprising data from 3 studies, with a total of 285 observations and 40 reported events related to serious adverse events, was conducted. The common effect model revealed a relative risk (RR) of 0.7666 (95% CI: 0.4323 to 1.3595), suggesting a potential reduction in serious adverse events, although this finding did not achieve statistical significance (z = − 0.91, p = 0.3632). There was no observed heterogeneity between the studies (I^2 = 0.0%, p = 0.9851). The funnel plot asymmetry test found no evidence of publication bias (t = 1.18, df = 1, p = 0.4464), indicating no evidence of publication bias. The bias estimate was 0.1542 (SE = 0.1302) (Fig. 7).

### Urticaria Adverse Events

**Fig. 8:**
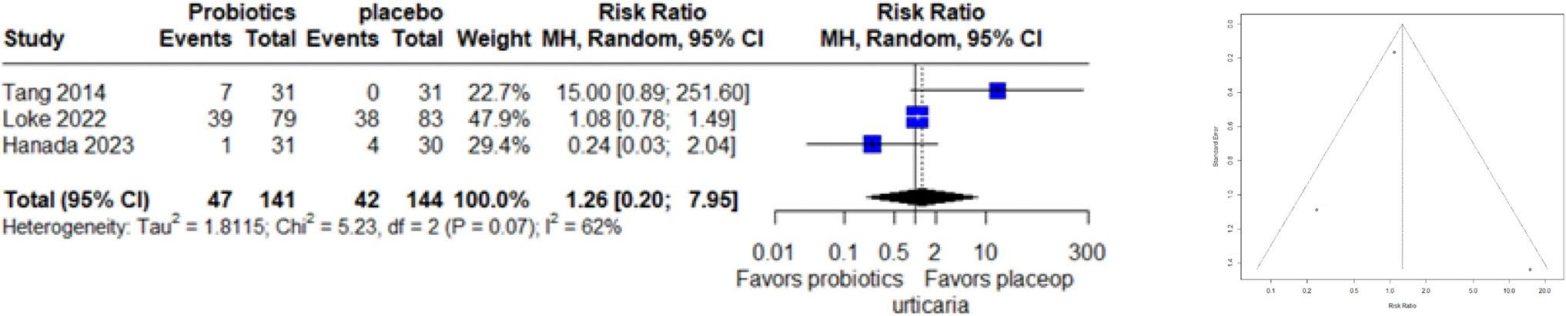
Forest plot (left) and funnel plot (right) of urticaria

A forest plot of three studies, involving 285 observations and 89 reported events related to urticaria adverse events, was conducted. The random-effects model revealed a relative risk (RR) of 1.2629 (95% CI: 0.2007 to 7.9479), indicating a slight increase in urticaria adverse events with probiotics compared to placebo, although this finding was not statistically significant (z = 0.25, p = 0.8036). Moderate heterogeneity was observed among the studies (I^2 = 61.8%, p = 0.0730), suggesting variability in effect sizes. The funnel plot asymmetry test found no evidence of publication bias (t = 0.17, df = 1, p = 0.8945). The bias estimate was 0.3131 (SE = 1.8725) (Fig. 8).

## Discussion

The evaluation of integrated probiotic therapy's safety profile revealed an overall favorable outcome, with no significant differences in adverse events compared to placebo for most organ systems examined.

This is explained by how probiotics exert their influence on the immune system. They stimulate the production of specific immune proteins that help decrease mucosal inflammation. Additionally, probiotics directly interact with the cells lining the intestines, triggering immunomodulative responses involving macrophages, T cells, and B cells [21]. These findings align with the hygiene theory, which posits that early childhood exposure to microorganisms plays a protective role against allergies and strengthens the immune system [22].

Studies investigating the use of probiotics as standalone treatments for immune response related diseases such as allergic rhinitis have demonstrated a reduction in rhinitis symptoms, underscoring their potential efficacy in managing allergic conditions [23]. Moreover, research has shown that probiotics can enhance the effectiveness of other pre-existing allergic treatments, such as cetirizine. In fact, the combination of cetirizine and probiotics led to a significant improvement in allergic rhinitis symptoms compared to cetirizine alone [24]. On the other hand, another study conducted on probiotics and asthma revealed no benefit in symptoms suggesting conflicting evidence exists regarding the effects of probiotics on allergic diseases, most likely due to a lack of comprehensive data [25].

Other treatments, such as anti-immunoglobulin E therapy, particularly omalizumab, have undergone testing for food allergies and should be considered alongside probiotics. Evidence indicates that omalizumab has a positive effect on food allergies and is safe for use as a standalone treatment [26]. Furthermore, when combined with oral immunotherapy, omalizumab does not increase adverse effects [27]. However, it is important to note that the cost of omalizumab is significantly higher compared to probiotics, which raises concerns about its cost-effectiveness in relation to its benefits [28]. Probiotics could potentially offer a more affordable alternative. Therefore, conducting studies that compare the efficacy of both treatments would be beneficial.

Despite the results suggesting probiotics are generally well-tolerated, it is important to note the potential increase in oral adverse effects. Although further research is required to establish statistical significance, this specific deviation from common results could be explained by host gut factors. For instance, one study revealed that the conditions present in the gastrointestinal tract can affect the activity of specific genes in certain *lactobacillus* strains. Factors such as acidified milk during gastric digestion or duodenal juice and bile can interfere with probiotic function [21]. Moreover, the particular strain of probiotic bacteria used could also provide different results. For example, *lactobacillus rhamnosus*, one of the strains examined in the study, appears to positively impact healing processes in the gut mucosa, which could explain why the adverse effects were not significantly different from the placebo group in GIT adverse effects analysis [21].

Additionally, a study assessing various strains of probiotics collectively did not demonstrate significant benefits, but subgroup analysis focusing on *lactobacillus rhamnosus* GG revealed positive outcomes [29]. Furthermore, *lactobacillus rhamnosus* induces immunomodulation in a dose-dependent manner, suggesting that different dosages may yield different results [21]. Other limitations were that the number of studies available for certain adverse events restricts the assessment of heterogeneity and publication bias, highlighting the need for further research in those specific areas. There are four phase 2 trials underway assessing probiotics as an adjuvant to immunotherapy for peanut and egg allergies. Results from these ongoing studies may provide more insight into the efficacy and adverse effects profile of probiotics as an oral immunotherapy adjuvant and may provide insight on the effects of different doses [30–33].

## Conclusion

Although no statistically significant difference was found between probiotics and placebo as allergen-specific immunotherapy adjuvants, the therapeutic combination appears to be generally safe and shows promise as a valuable method. This emphasizes the need to further explore their potential benefits for children with food allergies, particularly with the rising prevalence of atopic diseases. Probiotics, as an adjuvant treatment, could potentially improve response to allergen-specific immunotherapy and decrease the duration of treatment, thereby reducing dependence on medications and mitigating their associated side effects. Nevertheless, the usage of probiotics as an adjuvant to immunotherapy should be approached with caution, with careful selection and monitoring of patients to ensure optimal outcomes. Further studies with standardized probiotic strains and larger sample sizes are necessary to provide more robust evidence.

## Limitations

We acknowledge that this study has a few limitations. We could only include three studies due to the limited number of completed RCTs available on this topic. There was also a notable heterogeneity in the dosing regimens of the interventions across the included trials. In addition, due to the limited sample size in both intervention groups, the outcomes did not achieve a statistically significant relative risk. Furthermore, the limited number of studies prevented us from conducting Egger’s test for some of the outcomes.

## Supplementary Information

Below is the link to the electronic supplementary material.

Supplementary file1 (PDF 165 KB)

## Data Availability

No datasets were generated or analysed during the current study.

## Declarations

### Data Statement

The datasets used and/or analyzed during the current study are available from the corresponding author on reasonable request.
