# Supplementary material for: Safety Profile of Probiotics as an Adjuvant to Oral Immunotherapy for Food Allergies: A Meta Analysis of Randomized Controlled Trials
Source: Probiotics Antimicrob Proteins. 2025 May 5;18(1):812–21. doi: 10.1007/s12602-025-10544-z (PMC12999830; doi:10.1007/s12602-025-10544-z)

Safety Profile of Probiotics as an Adjuvant to Oral Immunotherapy for Food Allergies: A Meta Analysis of Randomized Controlled Trials

Supplemental Data

Table of Contents

Supplemental File 1: Search Strategy 2
Supplemental Table 1: Study Characteristics 4
Supplemental Table 2: Demographic Characteristics 9
Supplemental Table 3: Clinical Characteristics 11
Supplemental Figure 1: Risk of Bias Results 13

Search Strategy

Searches, Mesh Terms, And Synonyms Used to Interrogate the Data Sources

Date of Search: 3/1/2024

MeSH terms used:

Word	MeSH Terms and Synonyms
Probiotics	Probiotics<br>Lactobacillus<br>Bifidobacterium
Allergen-specific immunotherapy	Allergen-specific immunotherapy<br>AIT<br>Oral immunotherapy<br>Specific immunotherapy<br>SIT<br>Desensitization<br>Tolerance induction<br>SCIT<br>Sublingual immunotherapy
Food allergy	Food allergy<br>Peanut allergy<br>Milk allergy<br>Egg allergy<br>Soy allergy<br>Nut allergy<br>Shellfish allergy
Safety	Efficacy<br>Safety<br>Side effects<br>Symptom control<br>Quality of life<br>Remission<br>Treatment

Strategy: ((probiotics) OR (Lactobacillus) OR (Bifidobacterium)) AND ((allergen-specific immunotherapy) OR (AIT) OR (oral immunotherapy) OR (specific immunotherapy) OR (SIT) OR (desensitization) OR (tolerance induction) OR (SCIT) OR (sublingual immunotherapy)) AND ((food allergy) OR (peanut allergy) OR (milk allergy) OR (egg allergy) OR (soy allergy)

OR (nut allergy) OR (shellfish allergy)) AND ((efficacy) OR (safety) OR (side effects) OR (symptom control) OR (quality of life) OR (remission) OR (treatment))

Results:

PubMed: 154

Scopus: 246

WOS: 119

Supplemental Table 1: Characteristics of the Included Studies

Study ID	Title	Study design	Site	Timeframe	Allergen	Population (Inclusion criteria)	Length of follow-up	Primary outcome	Groups and sample size		Intervention	Probiotic Used
									Group	Sample size		
Tang et al. (2015) (18)	Administration of a probiotic with peanut oral immunotherapy: A randomized trial	double-blind, placebo-controlled rct	Australia	18.8 months	peanut	children aged 1-10 years with peanut allergy	22 months	desensitization to peanuts in 89.7% of the group with the intervention and 7.1% of the placebo group	probiotic and peanut oral immunotherapy	31	Lactobacillus rhamnosus CGMCC 1.3724 at a fixed dose of 2x10^10 colony-forming units (freeze-dried powder) once daily together with peanut OIT (peanut flour, 50% peanut protein)	Lactobacillus rhamnosus CGMCC 1.3724

									placebo probiotic and placebo peanut oral immunotherapy	31	placebo (maltodextrin) and placebo (maltodextrin, brown food coloring, and peanut essence) once daily	
Loke et al. (2022) (19)	Probiotic peanut oral immunotherapy versus oral immunotherapy and placebo in children with peanut allergy in Australia (PPOIT-003): a multicentre,	double-blind placebo-controlled rct	Australia	38.6 months	peanut	children aged 1–10 years, weighing more than 7 kg, with peanut allergy confirmed positive peanut skin prick test (≥3 mm) or peanut-specific IgE	12 months	PPOIT and OIT were effective at inducing sustained unresponsiveness	probiotic and peanut oral immunotherapy	79	peanut protein (commercially available food-grade 12% defatted peanut flour [50% peanut protein]) until a 2000 mg daily maintenance dose was	Lactobacillus rhamnosus ATCC 53103

	randomised, phase 2b trial					(≥0·35 kU/L)					reached. The probiotic adjuvant was a daily dose of 2 × 10^{10} colony-forming units of the probiotic Lactobacillus rhamnosus ATCC 53103.	
									placebo probiotic and peanut oral immunotherapy	83	probiotic placebo (maltodextrin) and peanut protein	

Yamato-Hanada et al. (2023) (20)	Combination of heat-killed Lactiplantibacillus plantarum YIT 0132 (LP0132) and oral immunotherapy in cow's milk allergy: a randomised controlled trial	double-blind placebo-controlled rct	Japan	6 months	cow milk	cow milk allergic children aged 1-18 years diagnosed by the oral-milk challenge test who had a plan to take oral immunotherapy for cow milk allergy; and voluntary willingness and ability to comply with the study requirements and	24 weeks	improved tolerance to cow milk	probiotic and cows milk oral immunotherapy	31	combination of heat-killed Lactiplantibacillus plantarum YIT 0132 (LP0132) in citrus juice and oral immunotherapy for treating IgE-mediated cow milk allergy	Lactiplantibacillus plantarum YIT 0132 (LP0132)
									placebo probiotic and cows milk oral immunotherapy	30	combination of placebo citrus juice without LP0132 and oral immunotherapy	

						procedures after obtaining written informed consent.						

Supplemental Table 2: Demographic Characteristics of Included Participants

Study ID	Age (mean)			sex					
	Probiotic + Immunotherapy	Placebo probiotic + Placebo immunotherapy	Oral immuntotherapy + placebo probiotic	female			male		
				Probiotic + Immunotherapy	Placebo probiotic + Placebo immunotherapy	Oral immuntotherapy + placebo probiotic	Probiotic + Immunotherapy	Placebo probiotic + Placebo immunotherapy	Oral immuntotherapy + placebo probiotic
Tang et al. (2015)	6.1	5.8		14	11		17	20	

Loke et al. (2022)	6		5.8	30		28	49		55
Yamamoto-Hanada et al. (2023)	5		5	7		13	24		17

Supplemental Table 3: Clinical Characteristics of Included Participants

Study ID	History of doctor-diagnosed eczema			History of doctor-diagnosed asthma			Anaphylaxis to peanut			multiple food allergy diagnosed			median peanut specific IgE kU/L)		
	Probiotic + Immunotherapy	Placebo probiotic + Placebo immunotherapy	Oral immunotherapy + placebo probiotic	Probiotic + Immunotherapy	Placebo probiotic + Placebo immunotherapy	Oral immunotherapy + placebo probiotic	Probiotic + Immunotherapy	Placebo probiotic + Placebo immunotherapy	Oral immunotherapy + placebo probiotic	Probiotic + Immunotherapy	Placebo probiotic + Placebo immunotherapy	Oral immunotherapy + placebo probiotic	Probiotic + Immunotherapy	Placebo probiotic + Placebo immunotherapy	Oral immunotherapy + placebo probiotic
Tang et al. (2015)	24	24		16	14		14	10					14.3	8.25	

Loke et al. (2022)	60		61	18		31	28		32	54		52	10.4		11
Yamamoto-Hanada et al. (2023)	23		20	12		14				25		23	n/a		

Supplemental Figure 1: Risk of Bias Results

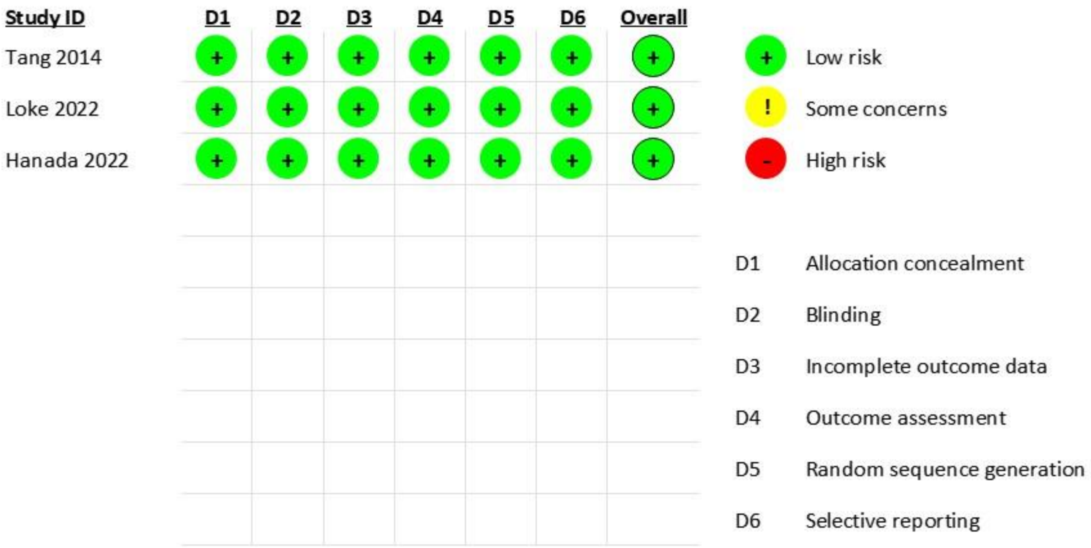

Supplement: Supplementary file 1 — Supplementary file1 (PDF 165 KB) [file 12602_2025_10544_MOESM1_ESM.pdf]
